# Pulmonary Arterial Hypertension and Adverse Outcomes after Kidney Transplantation: A Systematic Review and Meta-Analysis

**DOI:** 10.3390/jcm11071944

**Published:** 2022-03-31

**Authors:** Crischentian Brinza, Adrian Covic, Anca Elena Stefan, Mariana Floria, Iolanda Valentina Popa, Dragos-Viorel Scripcariu, Alexandru Burlacu

**Affiliations:** 1 Institute of Cardiovascular Diseases, 700503 Iasi, Romania; crischentian-branza@email.umfiasi.ro; 2 Faculty of Medicine, University of Medicine and Pharmacy Grigore T Popa, 700115 Iasi, Romania; adrian.covic@umfiasi.ro (A.C.); floria.mariana@umfiasi.ro (M.F.); iolanda-valentina.g.popa@umfiasi.ro (I.V.P.); 3 Nephrology Clinic, Dialysis and Renal Transplant Center—Constantin Ion Parhon University Hospital, 700503 Iasi, Romania; ancaelena-stefan@email.umfiasi.ro; 4 Doctor Iacob Czihac Military Emergency Clinical Hospital, 700483 Iasi, Romania

**Keywords:** pulmonary arterial hypertension, kidney transplantation, adverse outcomes, meta-analysis, prediction

## Abstract

Pulmonary arterial hypertension (PH) has a high prevalence in chronic kidney disease (CKD) patients, especially those undergoing kidney transplantation (KT). We aimed to systematically review and calculate the pooled effect size of the literature evaluating the association between pre-existing PH documented by transthoracic echocardiography (TTE) or invasively and adverse outcomes following KT. The primary composite outcome extracted from the included studies was represented by the mortality from any cause following KT and delayed graft function (DGF), graft dysfunction, or graft failure. The secondary outcomes were represented by individual components of the primary composite outcome. Twelve studies meeting the inclusion criteria were selected. The main finding is that pre-existing PH was associated with increased mortality and a higher rate of DGF, kidney graft dysfunction, or failure in KT recipients. The effect remained significant for all outcomes irrespective of PH evaluation, invasively or using TTE. Consequently, patients with PH defined only by TTE were at higher risk of death, DGF, or graft failure. Our findings support the routine assessment of PH in patients on the KT waitlist. PH might represent an extensively available and valuable tool for risk stratification in KT patients. These data should be confirmed in large prospective clinical trials.

## 1. Introduction

Chronic kidney disease (CKD) still exerts a significant social and healthcare burden, despite a lower trend towards years lived with disability, years of life lost, and disability-adjusted life-years [1]. Almost 700 million people were diagnosed with CKD until 2017. Although end-stage kidney disease (ESKD) prevalence was lower (0.07%) than stage 3 (3.9%) or stage 4 CKD (0.16%), it had the most significant impact on morbidity and mortality [1]. Moreover, more than half of patients with CKD stage 3 could progress to CKD stage 4 or ESKD during long-term follow-up, accentuating the burden of the disease [2].

Once CKD reaches ESKD, renal replacement therapy (RRT) is needed. Kidney transplantation (KT) is the first option of treatment in eligible individuals, being a cost-effective method of RRT as compared to dialysis [3,4]. Although it requires immunosuppressive therapy and close monitoring, KT offers a better quality of life compared to that of dialysis patients [5,6,7,8]. KT was also associated with a lower mortality risk than dialysis and a longer life expectancy [5,9].

Clinical studies reported that pulmonary arterial hypertension (PH) had a relatively high prevalence in CKD patients, especially those undergoing RRT. One meta-analysis reported a 30% prevalence of PH in patients with CKD, which was even higher in the ESKD subgroup (35%) [10]. In another study, the authors recorded a 34.6% incidence of PH in dialysis patients, which was concordant with results observed in the meta-analysis mentioned above [11]. These data highlight that PH does not represent a rare condition in ESKD patients, as it could affect more than a third of subjects.

Furthermore, PH in ESKD patients is associated with various worse outcomes. As previously documented, the subgroup of patients with CKD and PH displayed an increased all-cause and cardiovascular mortality (respectively, RR = 2.08 and 95% CI = 1.06–4.08, and RR = 3.77 and 95% CI = 2.46–5.78) [10]. A recent study with a larger sample size (30,052 CKD patients with PH) confirmed that PH was linked to a greater mortality risk during five years of follow-up (HR 1.47, 95% CI, 1.40–1.53). Besides mortality, PH patients were at an increased risk of hospitalizations, mainly due to cardiovascular causes (rate ratio 4.61) [12].

The potential impact of pre-existing PH on short- and long-term KT outcomes is of particular interest. Besides invasive measurement methods, PH could be easily assessed non-invasively by using transthoracic echocardiography (TTE) in a pre-KT setting [13]. Therefore, PH might represent an extensively available and valuable tool for risk stratification in KT patients.

Unfortunately, studies investigating pre-existing PH as a predictor for adverse events following KT are limited to observational data. Regarding the prognostic value of PH in KT candidates, one meta-analysis published in 2017 reported that patients with PH had a higher mortality risk than those without PH (OR 3.15, 95% CI, 1.42–6.97, *p* = 0.005). However, this paper included only three studies with a small sample analyzed (*n* = 502) [14]. Moreover, one included study did not specify if all KT candidates underwent KT during follow-up [15]. Consequently, the utility of pre-existing PH to promptly recognize high-risk KT recipients needs to be elucidated.

As new data became available in the past five years, we systematically reviewed the literature evaluating the association between pre-existing PH (documented either by TTE or invasively) and adverse outcomes following KT.

## 2. Materials and Methods

The updated Preferred Reporting Items for Systematic Reviews and Meta-Analyses (PRISMA) guidelines were applied to standardize data search, collection, synthesis, and reporting (Supplementary Table S1) [16]. The protocol was registered in PROSPERO database (CRD42022306978).

### 2.1. Data Sources and Search Strategy

Potentially relevant studies were searched in the following databases, from 10 December 2021 till 20 January 2022: MEDLINE (PubMed), Embase, Cochrane, and Scopus. Language filters were not applied in the search process. In addition to the sources mentioned above, Google Scholar and ClinicalTrials.gov databases were screened for additional citations. References from representative studies were also searched to retrieve further studies for eligibility assessment. We used different combinations of keywords and controlled vocabulary to create a comprehensive search strategy: “pulmonary hypertension”, “pulmonary pressure”, “echocardiography”, “kidney transplant”, “renal transplant”, “kidney graft”, “renal graft”, “outcomes”, “mortality”, “survival”, “kidney graft dysfunction”, “renal graft dysfunction”, “kidney graft survival”, and “renal graft survival”. The complete search strategy is described in Supplementary Table S2.

### 2.2. Eligibility Criteria and Outcomes

Two independent investigators decided to include eligible studies in the present systematic review based on several pre-specified inclusion and exclusion criteria. We established the following inclusion criteria before performing the search in the databases and data extraction: (1) studies with a randomized controlled or an observational design, (2) studies which included humans ≥ 18 years old, (3) population was represented by patients with KT, (4) PH was appraised non-invasively (using TTE) or invasively prior to KT, and (5) studies provided data regarding the association between PH and mortality or kidney graft dysfunction and failure. Furthermore, case reports, editorials, studies with overlapping populations, unpublished data, and meta-analyses were excluded. Moreover, studies with missing outcome data were excluded from the analysis. Any possible disagreements were solved by discussion and consensus.

The primary composite outcome included mortality from any cause following KT, and delayed graft function (DGF), graft dysfunction, or graft failure. The secondary outcomes were represented by individual components of the primary composite outcome, respectively, any-cause mortality and delayed graft function, graft dysfunction, or graft failure.

### 2.3. Data Collection and Synthesis

After eligibility assessment and inclusion of studies in the present systematic review, the following data were extracted by two independent investigators: first author, publication year, study design, number of patients included, age, the definition of pulmonary hypertension used, clinical setting, and comorbidities, investigated outcomes and number of events, and follow-up duration.

The pooled effect size, respectively odds ratio (OR), and corresponding 95% confidence intervals (CIs) were obtained by using Review Manager (RevMan) version 5.4.1 (Nordic Cochrane Centre, The Cochrane Collaboration, 2020, Copenhagen, Denmark). For this purpose, the random-effect model and Mantel–Haenszel method were used in dichotomous data. The heterogeneity of included studies was assessed by using I^2^ statistics, as follows: 0–25% (low), 26–50% (moderate), 51–75% (high) and >75% (very high). A *p*-value lower than the threshold of 0.05 was considered to be significant.

Sensitivity analysis was performed by sequentially excluding studies which evaluated PH invasively versus those investigating PH non-invasively, as well as studies that analyzed specific outcomes, mortality versus graft dysfunction or failure.

### 2.4. Quality Assessment

Newcastle–Ottawa Scale (NOS) was used to evaluate the overall quality of non-randomized studies [17]. NOS represents a star-based grading system consisting of three domains: selection, comparability of groups, and investigated outcomes. Each domain encompasses a set of crucial questions, for which stars are designated according to studies’ quality judgment [17].

## 3. Results

**Figure 1:**
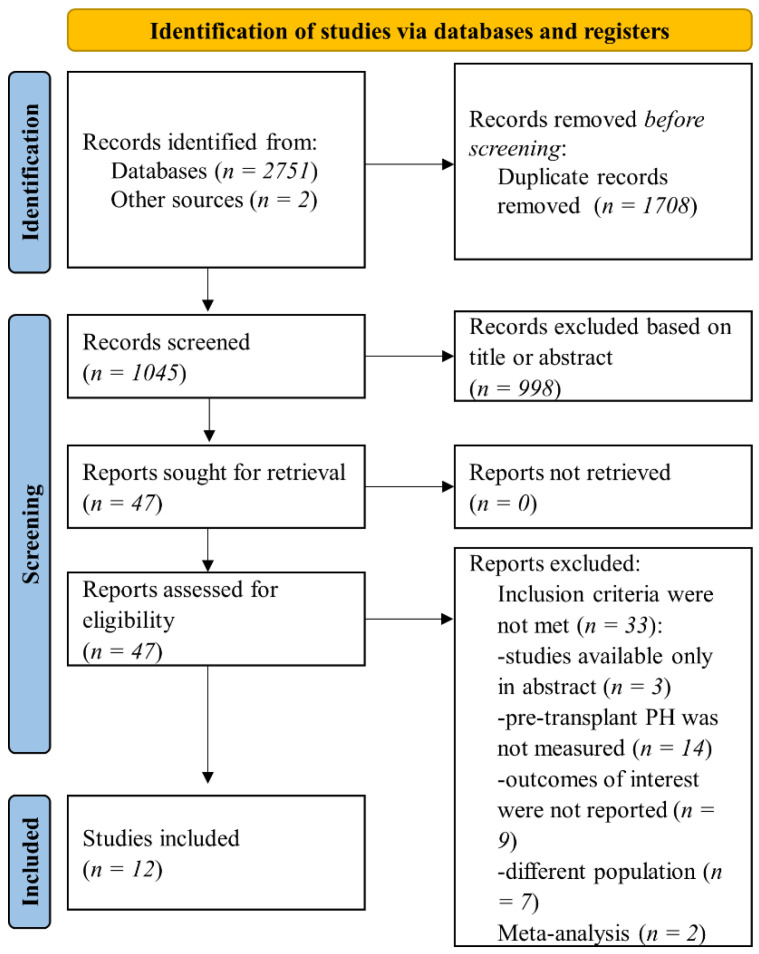
Flow diagram of selected studies in present analysis.

A search was performed in the databases mentioned above, and we retrieved 2751 records. Duplicate publications were excluded, leaving 1045 works to be further assessed for the eligibility criteria. After additional exclusion of studies based on title and abstract screening, two independent investigators evaluated full-text articles for inclusion and exclusion criteria. In the final analysis, 12 studies were included, as was presented in the search flowchart (Figure 1).

General data (study design, number of patients included, age of participants, PH definition used, investigated outcomes, and follow-up period) and results reported in analyzed studies were presented in Table 1 and Table 2, respectively.

All included studies were observational and had a retrospective design [18,19,20,21,22,23,24,25,26,27,28,29]. Most studies evaluated echocardiographic parameters as indirect markers of PH [18,20,21,22,23,24,25,26,27,28,29], while only one study included right heart catheterization in PH diagnostic algorithm [19]. PH was defined by using pulmonary artery systolic pressure (PASP) estimated by TTE in six studies [20,22,23,24,25,27]. Right ventricular systolic pressure (RVSP), maximum tricuspid regurgitation jet velocity (TRJV), and right ventricular dilation and function were other TTE parameters used in clinical trials to estimate PH [18,21,26,28,29].

The primary outcomes investigated in clinical studies were mortality, DGF, graft failure, graft dysfunction, or graft loss. DGF was defined as a dialysis requirement in the first seven days following KT [19,25]. One study also included creatinine ≥ 3 mg/dL on day 5 after transplant for DGF definition [27]. The follow-up period varied across studies, from the early postoperative [23] to more than 9 years [29].

**Figure 2:**
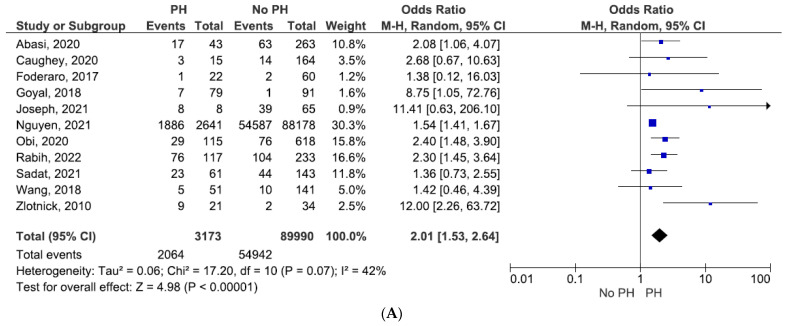

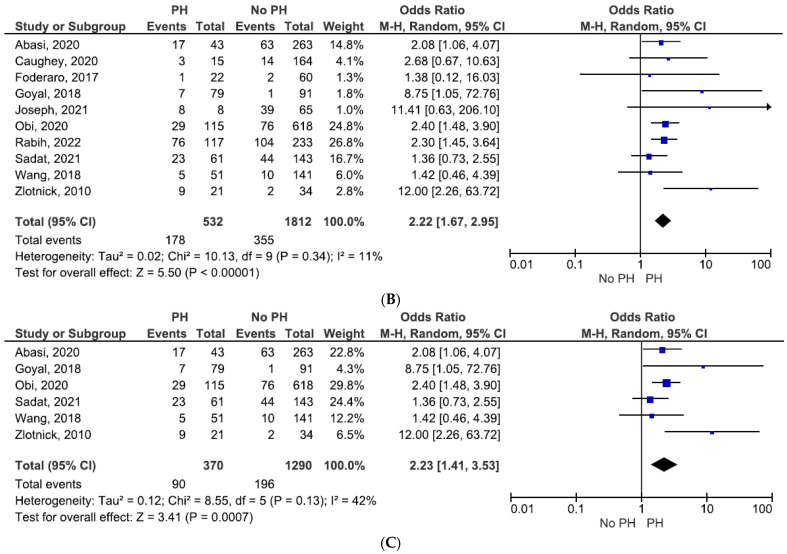
(**A**) Primary composite outcome (mortality from any cause following KT and delayed graft function (DGF), graft dysfunction, or graft failure). (**B**) Primary composite outcome estimated by TTE only. (**C**) Primary composite outcome estimated by PASP–TTE.

In the pooled analysis, the primary composite outcome was observed in 2064 patients with KT and PH, compared to 54,942 patients with KT, but no documented signs of PH. Patients with PH exhibited a two-fold higher risk of primary composite outcome occurrence (OR = 2.01, 95% CI = 1.53–2.64, and *p* < 0.00001; see Figure 2A), with moderate heterogeneity (I^2^ = 42%). In order to investigate the impact of PH assessed non-invasively by TTE, we excluded the study which incorporated right heart catheterization in PH definition [19].

The effect of PH estimated by TTE on the primary composite outcome remained statistically significant (OR = 2.22, 95% CI = 1.67–2.95, and *p* < 0.00001; see Figure 2B), with lower heterogeneity (I^2^ = 11%).

Furthermore, we analyzed the effect of PH defined only non-invasively by PASP. We also observed a significantly higher risk in the case of patients with PH as compared to those without PH (OR 2.23, 95% CI, 1.41–3.53, and *p* = 0.0007; see Figure 2C).

**Figure 3:**
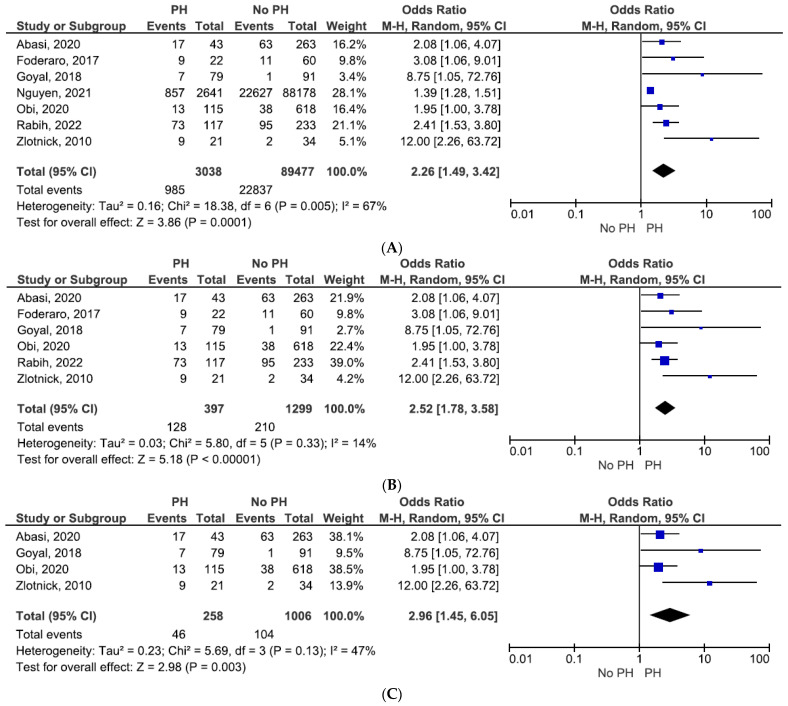
(**A**) Graft dysfunction or failure. (**B**) Graft dysfunction or failure estimated by TTE only. (**C**) Delayed graft function or failure estimated by PASP–TTE only.

In addition, we evaluated individual components of the primary composite outcomes concerning pre-existing PH. Patients with KT and documented PH prior to surgery had an increased risk of DGF, graft dysfunction, or failure (OR = 2.26, 95% CI = 1.49–3.42, and *p* = 0.0001; see Figure 3A).

The effect maintained significant regardless of the PH definition used. In the pooled analysis of studies that estimated PH by TTE, PH was associated with a greater risk of DGF, graft dysfunction, or failure (OR = 2.52, 95% CI = 1.78–3.58, and *p* < 0.00001; see Figure 3B).

Furthermore, PH evaluated only by PASP was linked to a higher risk of kidney graft dysfunction or failure (OR = 2.96, 95% CI = 1.45–6.05, and *p* = 0.003; see Figure 3C).

**Figure 4:**
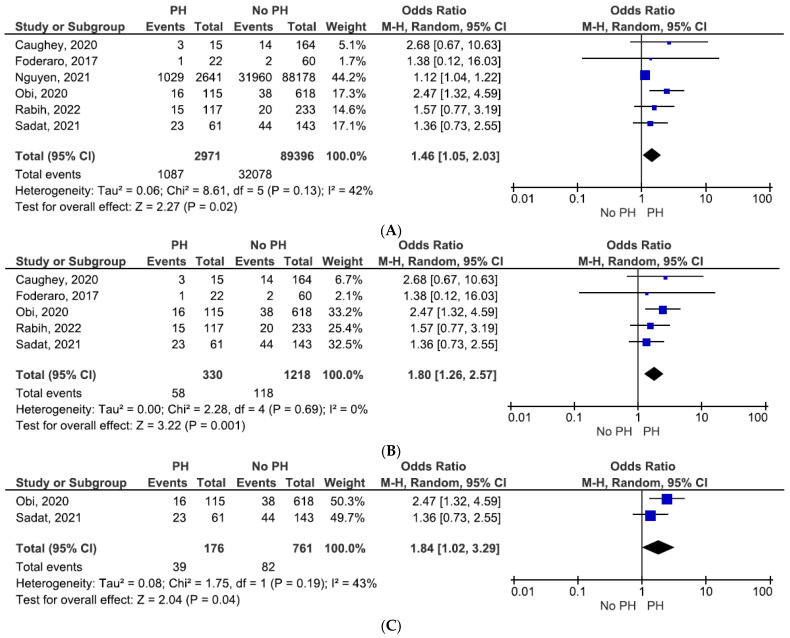
(**A**) Mortality. (**B**) Mortality estimated by TTE only. (**C**) Mortality estimated by PASP–TTE only.

Six studies reporting mortality events in KT recipients were analyzed (Figure 4). Patients with PH documented by TTE or invasively were associated with an increase in mortality risk after surgery (OR = 1.46, 95% CI = 1.05–2.03, and *p* = 0.02; see Figure 4A), with moderate heterogeneity across studies (I^2^ = 42%).

Mortality was significantly higher in PH patients (OR = 1.80, 95% CI = 1.26–2.57, and *p* = 0.001), even after exclusion of the study focused on invasive PH measurement (Figure 4B) [19].

Only two studies evaluated PH as PASP by using TTE and reported data regarding mortality events. Nevertheless, mortality steadily increased (OR 1.84, 95% CI, 1.02–3.29, *p* = 0.04) in patients with PH measured by PASP (Figure 4C).

Even though the global effect of PH on mortality was significant, some data from individual studies were discrepant. One study documented increased mortality only at the 5-year follow-up, but not during the first three years after KT. In addition, at the time of the multivariate analysis, the impact of pre-existing PH on KT recipients’ mortality was not significant; it was not at the 5-year follow-up, either [20]. The authors from another study did not report any differences in the 5-year survival rate in KT patients with PH or those without PH (*p* = 0.2). Nonetheless, the composite of death, graft dysfunction, or failure was significantly higher in PH patients who underwent KT (*p* = 0.001) when compared to those without PH [21].

Data regarding DGF or graft failure were also discordant in clinical studies. PH assessed invasively prior to KT was linked to an increased DGF, as observed in one study [19]. PH evaluated by using TTE was associated with a higher risk of DGF and early graft dysfunction in some studies, as well [25,27]. However, other studies did not reveal any statistically significant association between pre-existing PH and graft failure (*p* = 0.7) [21] or DGF.

**Figure 5:**
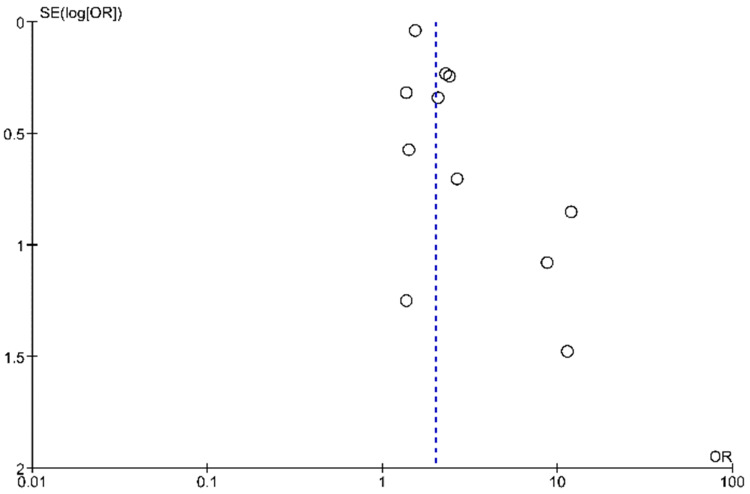
Funnel plot.

Although all included studies were observational, the overall quality was evaluated to be fair to good, as appraised by using NOS for non-randomized studies (Supplementary Table S3). Publication bias was evaluated and presented as a funnel plot (Figure 5).

## 4. Discussion

Our meta-analysis provides updated information, with the latest clinical studies addressing the utility of PH assessment in adverse outcomes prediction following KT.

The main finding of the present systematic review and meta-analysis is that pre-existing PH was associated with increased mortality and a higher rate of delayed graft function, kidney graft dysfunction, or failure in KT recipients. The effect remained significant for all outcomes irrespective of PH evaluation, invasively or using TTE. Consequently, patients with PH defined only by TTE were at higher risk of death, DGF, or graft failure. Although right heart catheterization represents the gold standard for establishing the diagnosis of PH, TTE markers, such as PASP, RVSP, and TRJV, could be feasible alternatives to predict adverse events in KT patients.

Early data from 2008 [18] and 2010 [25] showed that patients with pre-existing PH displayed an increased risk of mortality and DGF. The effect on mortality was independent in relation to other risk factors (age, left ventricular ejection fraction, and serum albumin), which represented a baseline and a motivation for future studies [18]. Since then, more evidence has become available, but data were sometimes discrepant. Therefore, a quantitative analysis is required to establish the potential predictive value of PH for adverse outcomes following KT.

The most used parameter for PH definition in analyzed studies was PASP evaluated by TTE. However, PASP cutoff values varied among included studies. Most authors used the 35 mmHg PASP cutoff to discriminate patients with or without PH [20,23,25,27], while the others adopted different values, 40 mmHg [22] and 37 mmHg [24], respectively. Hence, standardized cutoff values of TTE parameters and measurement methods are required to obtain generalizable and reproducible results in future clinical studies.

Furthermore, PASP, RVSP, and TRJV were also valuable markers of PH, but cutoff values were also subjugated to variance. Accordingly, RVSP > 50 mmHg, >40 mmHg or ≥35 mmHg cutoffs were used to identify PH patients [18,21,28]. Thus, we also performed an analysis of clinical studies that investigated only PASP to reduce the heterogeneity of PH assessment methods. Nevertheless, the presence of PH (defined on PASP) was associated with an increased risk of primary composite outcome and individual secondary outcomes (mortality, DGF, or kidney graft failure).

Notably, pre-existing PH, but not left ventricular systolic or diastolic dysfunction, was associated with worse outcomes in KT patients in multivariable analysis (respectively, RR = 1.432, 95% CI = 1.189–1.724, and *p* < 0.001; and RR = 1.031, 95% CI = 0.844–1.258, and *p* = 0.767) [21]. These data highlight the importance of PH evaluation in patients waiting for KT more than other echocardiographic parameters do.

In addition to specified primary and secondary outcomes, one study investigated the association between PH and perioperative complications in KT patients [23]. Patients with PH developed perioperative hypotension more frequently than patients without PH (26.6% vs. 9.9%, *p* = 0.004), and this imposed hemodynamic support. However, other perioperative complications, such as arrhythmias, myocardial infarction, pulmonary edema, and atelectasis, had similar incidences among KT patients, irrespective of PH presence [23].

Though pre-existing PH was associated with worse outcomes during short- and long-term follow-up, it should not be regarded as a contraindication for KT policies. One study reported that KT in PH patients improved survival rate compared to patients on the waitlist who did not undergo KT (respectively, 70.9% and 53% at five years). Moreover, mortality was 46% lower after KT than in the case of patients waiting for KT and pre-operatively documented PH [19]. For that reason, PH assessment prior to KT should not prohibit surgical intervention. Nevertheless, PH could be introduced in clinical practice as a risk marker for future adverse events in this population.

In addition, clinical outcomes following KT could vary concerning PH etiology. Though data are lacking in this particular context, it could be extrapolated from the general population. In this regard, one study observed that patients with left ventricular systolic dysfunction and valvular incompetence exhibited a higher mortality risk when compared to patients without documented left heart disease (HR = 1.30, 95% CI = 1.20–1.42, and *p* < 0.0001) [30]. Therefore, we need more accurate data regarding PH etiology and associated factors linked with poor outcomes; such data represent a direction of further research.

Several limitations in the current meta-analysis should be addressed. All included studies had a non-randomized, observational, and retrospective design, so data should be interpreted cautiously. Moreover, the PH definition, TTE parameters used, and cutoff values applied varied in clinical studies, contributing to an increased heterogeneity. In addition, few studies reported the prevalence of risk factors such as donor type (living versus deceased) or cold ischemia time in both PH and no-PH groups of patients; thus, they were not included in the analysis. Moreover, studies did not report the timing of PH assessment in relation to fluid status and hemodialysis, so this might impact TTE parameters. More prospective randomized controlled clinical trials with a large sample size that address the mentioned limitations are required to confirm these findings.

## 5. Conclusions

In the present meta-analysis, we observed that pre-existing PH (assessed either by TTE or invasively) was associated with an increased risk of all-cause mortality, DGF, kidney graft dysfunction, or failure following KT. Notably, patients with PH defined only by TTE or PASP were at a higher risk of worse outcomes during follow-up. Currently used TTE markers of PH, such as PASP, RVSP, and TRJV, could be used to predict adverse events in KT recipients in the pre-surgical setting. While PH identification must not hinder KT indication, patients with pre-existing PH might benefit from a closer perioperative and long-term follow-up monitoring to timely diagnose potential complications. Our findings support the routine assessment of PH in patients on the waitlist for KT, but these data should be confirmed in large prospective clinical trials.

## Figures and Tables

**Table 1:** General characteristics of studies included in present systematic review and meta-analysis.

Author, Year	Design	Patients, No.	Age, Median/Mean ± SD	Setting	Parameters Evaluated	Methods	Outcomes	Follow-Up Period
Issa et al., 2008 [18]	Observational, single-center, retrospective	215	55 ± 11	Adult KT recipients between January 2004 and June 2007 who had a pre-transplant TTE	RVSP LVEF LVH	PH was estimated by RVSP, using TTE: <35 mmHg (normal range), 35–50 mmHg (mild to moderate PH) and >50 mmHg (severe PH)	Primary endpoint: patient death with a functioning kidney graft	22.8 ± 11.8 months
Nguyen et al., 2021 [19]	Observational, multicenter (centers for Medicare and Medicaid Services), retrospective	90,819	52.5 ± 13.5 (without PH)	First-time adult KT recipients (between 2000 and 2016) reported by the US Renal Data System	PH	PH defined by a 2-component algorithm, including right heart catheterization	(a) Delayed graft function (dialysis within 7 days after transplant) (b) Death-censored graft failure (c) Mortality	4.3 years (with PH)
			55.7 ± 12.1 (with PH)					6.7 years (without PH)
Obi et al., 2020 [20]	Observational, single-center, retrospective	733	49.0 (without PH)	Adult KT patients between 2010 and 2015 who had a pre-transplant TTE	PASP LVEF Right atrial pressure	PH was estimated by using TTE: PASP < 35 mmHg (patients without PH) or PASP ≥ 35 mmHg (patients with PH)	(a) Mortality (b) Graft failure (c) Composite outcome of mortality or graft failure	46.9 months (without PH)
			56.0 (with PH)					36.9 months (with PH)
Rabih et al., 2022 [21]	Observational, single-center, retrospective	350	51.0 (without PH)	Adult KT recipients at Emory Transplant Center between 2010 and 2011 who had a pre-transplant TTE	RVSP TRJV LV systolic or diastolic dysfunction	PH was defined as RVSP ≥ 35 mmHg and/or maximum TRJV ≥ 2.9 m/s, as measured by TTE	(a) All-cause mortality (b) Graft dysfunction (stable creatinine ≥ 1.4 mg/dL) (c) Graft failure (requiring dialysis or retransplant)	5 years
			52 (with PH)					
Sadat et al., 2021 [22]	Observational, single-center, retrospective	204	–	Adult KT patients from 2010 to 2016 who had a pre-transplant TTE	PASP LVEF	PH was estimated by using TTE: PASP ≥ 40 mmHg (patients with PH) or PASP < 40 mmHg (patients without PH)	(a) Mortality (b) Graft function	77.9 ± 36.12 months
Goyal et al., 2018 [23]	Observational, single-center, retrospective	170	36.2 ± 11.2 (without PH)	Adult KT recipients who underwent a pre-transplant TTE examination	PASP LVEF	PH was estimated by using TTE: PASP ≥ 35 mmHg (patients with PH) or PASP < 35 mmHg (patients without PH)	(a) Primary outcome: delayed graft function (dialysis within 7 days after transplant) (b) Secondary outcomes: perioperative complications (hypotension, arrhythmias, need of post-operative mechanical ventilation, atelectasis, pulmonary edema)	–
			35.7 ± 9.8 (with PH)					
Wang et al., 2018 [24]	Observational, single-center, retrospective	192	50.3 ± 12.9 (without PH)	Consecutive adult KT recipients between 2008 and 2015 who had a pre-transplant TTE	PASP LVEF Right ventricular systolic function	PH was estimated by using TTE: PASP ≥ 37 mmHg (patients with PH) or PASP < 37 mmHg (patients without PH)	(a) Length of hospital stay after transplant (b) Renal allograft function by 1 and 2 years (creatinine and eGFR measurement at 1 and 2 years) (c) Recipient mortality (d) Rate of graft loss	4.0 ± 1.9 years
			52.7 ± 10.8 (with PH)					
Zlotnick et al., 2010 [25]	Observational, single-center, retrospective	55	52.4 ± 9.9 (without PH)	Adult KT recipients over a period of 3 years who had a pre-transplant TTE	PASP	PH was estimated by using TTE: PASP ≥ 35 mmHg (patients with PH) or PASP < 35 mmHg (patients without PH)	Early graft dysfunction: delayed graft function (dialysis within 7 days after transplant) or slow graft function (creatinine ≥ 3 mg/dL on day 5 after transplant, without dialysis)	–
			54.6 ± 13.2 (with PH)					
Caughey et al., 2020 [26]	Observational, retrospective	778 (179 KT recipients)	56.0 ± 10 (without PH)	Adult patients with advanced CKD included in the University of North Carolina Cardiorenal Registry	TRJV LVEF LVH Left atrial pressure	PH was estimated by using TRJV: ≥2.9 m/s ± other signs (interventricular septal flattening, dilated inferior vena cava)	Mortality	4.4 years
			57 ± 12 (with PH)					
Abasi et al., 2020 [27]	Observational, single-center, retrospective	306	37.33 ± 10.92 (without PH)	Adult KT recipient over a period of 4 years who had a pre-transplant TTE	PASP	PH was estimated by using TTE: PASP ≥ 35 mmHg (patients with PH) or PASP < 35 mmHg (patients without PH)	Delayed graft function (dialysis within the first week after transplant or creatinine ≥ 3 mg/dL on day 5 after transplant)	–
			35.26 ± 10.3 (with PH)					
Foderaro et al., 2017 [28]	Observational, single-center, retrospective	82	48.0 (without PH)	First-time adult KT recipients between 2003 and 2009 who had a pre-transplant TTE	RVSP LVEF	PH was estimated by using TTE: RVSP ≥ 40 mmHg (patients with PH)	(a) Death-censored allograft failure (b) Mortality	3 years
			50.0 (with PH)					
Joseph et al., 2021 [29]	Observational, single-center, retrospective	80 (RV function assessed in 73 patients)	51.3 ± 14.2	Adult KT recipients between 2008 and 2010 who had a TTE within 1 year prior to surgery	RV dilation and dysfunction LVEF	RVS dilation and function was established using TTE and standardized cutoff values	Primary outcome: composite of delayed graft function, graft failure, and all-cause mortality	9.4 ± 0.8 years

Note: eGFR = estimated glomerular filtration rate; KT = kidney transplant; LV = left ventricle; LVEF = left ventricular ejection fraction; LVH = left ventricular hypertrophy; PASP = pulmonary artery systolic pressure; PH = pulmonary hypertension; RV = right ventricle; RVSP = right ventricular systolic pressure; TRJV = tricuspid regurgitation jet velocity; TTE = transthoracic echocardiography.

**Table 2:** Results reported in studies included in present systematic review and meta-analysis.

Author, Year	Parameters	Outcomes	Results	
Issa et al., 2008 [18]	RVSP > 50 mmHg	Reduced recipient survival	HR 3.75 (95% CI, 1.17–11.97)	*p* = 0.016
Nguyen et al., 2021 [19]	PH	Delayed graft function	OR 1.23 (95% CI, 1.10–1.36)	*p* < 0.001
		Mortality	HR 1.56 (95% CI, 1.44–1.69)	*p* < 0.001
		Death-censored graft failure	HR 1.23 (95% CI, 1.11–1.38)	*p* < 0.001
Obi et al., 2020 [20]	PASP ≥ 35 mmHg		Univariate analysis:	
		Mortality (1 year)	HR 1.16 (95% CI, 0.33–4.04)	*p* = 0.82
		Mortality (3 years)	HR 1.71 (95% CI, 0.84–3.47)	*p* = 0.14
		Mortality (5 years)	HR 1.98 (95% CI, 1.11–3.56)	*p* = 0.02
		Composite of death or graft loss (5 years)	HR 1.69 (95% CI, 1.03–2.78)	*p* = 0.04
			Multivariate analysis:	
		Mortality (5 years)	HR 1.26 (95% CI, 0.66–2.41)	*p* = 0.49
		Graft failure (5 years)	HR 0.77 (95% CI, 0.31–1.91)	*p* = 0.57
Rabih et al., 2022 [21]	RVSP ≥ 35 mmHg and/or TRJV ≥ 2.9 m/s	Death, graft dysfunction, or failure	RR 1.432 (95% CI, 1.189–1.724)	*p* < 0.001
	LV systolic dysfunction	Death, graft dysfunction, or failure	RR 0.672 (95% CI, 0.347–1.302)	*p* = 0.239
	LV diastolic dysfunction	Death, graft dysfunction, or failure	RR 1.073 (95% CI, 0.824–1.399)	*p* = 0.600
Sadat et al., 2021 [22]	PASP ≥ 40 mmHg	Mortality	30.7% in patients without PH vs. 37.7% in patients with PH	*p* = 0.334
Goyal et al., 2018 [23]	PASP ≥ 35 mmHg	Delayed graft function	OR 8.75 (95% CI, 1.05–72.75)—univariate analysis	*p* = 0.017
			On multivariate analysis PH was not associated with delayed graft function	
Wang et al., 2018 [24]	PASP ≥ 37 mmHg	Death or graft loss (>2 years)	7.090% in patients without PH vs. 9.800% in patients with PH	*p* = 0.536
		Mean eGFR (2 years)	60.28 mL/min ± 20.94 in patients without PH vs. 51.04 ± 15.07 in patients with PH	*p* = 0.006
Zlotnick et al., 2010 [25]	PASP ≥ 35 mmHg	Early graft dysfunction	OR 15.0 (95% CI, 1.2–188.9)—adjusted for multiple variables	*p* = 0.03
			AUROC 0.74 (95% CI, 0.58–0.91)	
Caughey et al., 2020 [26]	TRJV: ≥2.9 m/s ± other signs	Mortality	8% in patients without PH and normal left atrial pressure vs. 17% in patients with PH with normal left atrial pressure	
Abasi et al., 2020 [27]	PASP ≥ 35 mmHg	Delayed graft function	39.5% in patients with PH vs. 24% in patients without PH	*p* < 0.05
Foderaro et al., 2017 [28]	RVSP ≥ 40 mmHg	Death-censored allograft failure	Three-fold higher risk in PH group (95% CI, 1.20–7.32)	*p* = 0.02
		Mortality	5% in patients with PH vs. 3% in patients without PH	*p* = 0.80
Joseph et al., 2021 [29]	RV dilation and dysfunction	Composite of delayed graft function, graft failure and all-cause mortality	100% in patients with RV dysfunction vs. 60% in patients without RV dysfunction	

Note: AUROC = the area under the receiver operating characteristic; eGFR = estimated glomerular filtration rate; LV = left ventricle; PASP = pulmonary artery systolic pressure; PH = pulmonary hypertension; RV = right ventricle; RVSP = right ventricular systolic pressure; TRJV = tricuspid regurgitation jet velocity.

## Data Availability

Not applicable.

## Supplementary Materials

The following supporting information can be downloaded at https://www.mdpi.com/article/10.3390/jcm11071944/s1. Table S1: PRISMA checklist. Table S2: Databases and search strategies used in present systematic review and meta-analysis. Table S3: Quality assessment of included studies, using Newcastle–Ottawa scale. File S1: PROSPERO registration (CRD42022306978).
