# Supplementary material for: Pulmonary Arterial Hypertension and Adverse Outcomes after Kidney Transplantation: A Systematic Review and Meta-Analysis
Source: J Clin Med. 2022 Mar 31;11(7):1944. doi: 10.3390/jcm11071944 (PMC8999673; doi:10.3390/jcm11071944)
Supplement: Supplementary file 1 [file jcm-11-01944-s001.zip › File S1. PROSPERO registration (CRD42022306978).pdf]

To enable PROSPERO to focus on COVID-19 submissions, this registration record has undergone basic automated checks for eligibility and is published exactly as submitted. PROSPERO has never provided peer review, and usual checking by the PROSPERO team does not endorse content. Therefore, automatically published records should be treated as any other PROSPERO registration. Further detail is provided here.

Citation

Crischentian Brinza, Anca-Elena Stefan, Iolanda Valentina Iolanda Valentina Popa, Adrian Covic, Alexandru Burlacu. Non-invasive pulmonary hypertension assessment for adverse outcomes prediction following kidney transplant: a systematic review and meta-analysis.. PROSPERO 2022 CRD42022306978 Available from: https://www.crd.york.ac.uk/prospero/display_record.php?ID=CRD42022306978

Review question

We aim to systematically review the literature to evaluate the association between pulmonary hypertension documented using transthoracic echocardiography and adverse outcomes following kidney transplant.

Searches

The search will be performed in the following databases, from the inception until 30th January 2022: MELDINE (PubMed), Embase, Cochrane and Scopus. Language filters will not be applied in the search process. In addition to aforementioned sources, Google Scholar and ClinicalTrials.gov databases will be screened for additional citations.

Types of study to be included

In our systematic review, both, randomized controlled trials (if available) and observational studies will be included.

Condition or domain being studied

We will evaluate the impact of pulmonary hypertension on adverse outcomes following kidney transplant, including all-cause mortality and graft dysfunction or failure.

Participants/population

We will include in our analysis patients ? 18 years old who underwent kidney transplant.

Intervention(s), exposure(s)

We will consider studies for inclusion if pulmonary hypertension was appraised non-invasively, prior to kidney transplant, using transthoracic echocardiography.

Comparator(s)/control

We will investigate the impact of pulmonary hypertension on outcomes following kidney transplant in comparison to patients without documented pulmonary hypertension prior to kidney transplant.

Context

Case reports, editorials, studies with overlapping population, unpublished data and meta-analyses will be excluded. Also, studies with missing outcome data will be excluded from the analysis.

Main outcome(s)

The association between pulmonary hypertension and mortality from any cause following kidney transplant and kidney graft dysfunction or failure will be investigated.

Additional outcome(s)

None

Data extraction (selection and coding)

After eligibility assessment and inclusion of studies in the present systematic review, the following data will be extracted by two independent investigators: first author, publication year, study design, number of

patients included, age, definition of pulmonary hypertension used, clinical setting and comorbidities, investigated outcomes and number of events, and follow-up duration.

Risk of bias (quality) assessment

Newcastle-Ottawa scale (NOS) will be used to evaluate the overall quality of non-randomized studies. NOS represents a star-based grading system, consisting of three domains: selection, comparability of groups and investigated outcomes. Each domain encompasses a set of key questions, for which stars are designated according to studies' quality judgement. Risk of bias of randomized trials (if available) will be assessed using the revised Cochrane risk-of-bias tool for randomized trials (RoB 2).

Strategy for data synthesis

When possible, data will be reported as numbers, intervals, percentages, risk ratio (RR), odds ratio (OR), median and mean values, confidence intervals and P values. Also, data will be presented as tables and figures. Following the extraction process, we will qualitatively and quantitatively (if applicable) synthetize the data.

Analysis of subgroups or subsets

We will investigate the impact of pulmonary hypertension on outcomes following kidney transplant in comparison to patients without documented pulmonary hypertension prior to kidney transplant.

Contact details for further information

Crischentian Brinza
cristian03_30@yahoo.com

Organisational affiliation of the review

Institute of Cardiovascular Diseases “ Prof. Dr. George I.M. Georgescu,” 700503, Iasi, Romania

Review team members and their organisational affiliations

Dr Crischentian Brinza. Institute of Cardiovascular Diseases “ Prof. Dr. George I.M. Georgescu,” 700503, Iasi, Romania
Dr Anca-Elena Stefan. Hospital
Dr Iolanda Valentina Iolanda Valentina Popa.
Professor Adrian Covic.
Professor Alexandru Burlacu.

Type and method of review

Meta-analysis, Prognostic, Systematic review

Anticipated or actual start date

25 January 2022

Anticipated completion date

31 March 2022

Funding sources/sponsors

This research received no external funding.

Conflicts of interest

Language

English

Country

Romania

Stage of review

Review Ongoing

Subject index terms status

Subject indexing assigned by CRD

Subject index terms

MeSH headings have not been applied to this record

Date of registration in PROSPERO

25 February 2022

Date of first submission

25 January 2022

Details of any existing review of the same topic by the same authors

No existing review

Stage of review at time of this submission

Stage	Started	Completed
Preliminary searches	Yes	No
Piloting of the study selection process	No	No
Formal screening of search results against eligibility criteria	No	No
Data extraction	No	No
Risk of bias (quality) assessment	No	No
Data analysis	No	No

The record owner confirms that the information they have supplied for this submission is accurate and complete and they understand that deliberate provision of inaccurate information or omission of data may be construed as scientific misconduct.

The record owner confirms that they will update the status of the review when it is completed and will add publication details in due course.

Versions

25 February 2022
25 February 2022
