# Supplementary material for: Pulmonary Arterial Hypertension and Adverse Outcomes after Kidney Transplantation: A Systematic Review and Meta-Analysis
Source: J Clin Med. 2022 Mar 31;11(7):1944. doi: 10.3390/jcm11071944 (PMC8999673; doi:10.3390/jcm11071944)
Supplement: Supplementary file 1 [file jcm-11-01944-s001.zip › Table S2 Databases and search strategy.pdf]

Table S2. Databases and search strategies used in present systematic review and meta-analysis.

Database	Coverage	Search run	Records
**MEDLINE**	1946 to present	"((pulmonary hypertension) OR (pulmonary pressure)) AND "Kidney Transplantation"[Mesh] AND ((outcomes) OR (mortality) OR (survival) OR (kidney graft dysfunction) OR (renal graft dysfunction) OR (kidney graft survival) OR (renal graft survival))"	185
		"(echocardiography) AND ((pulmonary hypertension) OR (pulmonary pressure)) AND "Kidney Transplantation"[Mesh] AND ((outcomes) OR (mortality) OR (survival) OR (kidney graft dysfunction) OR (renal graft dysfunction) OR (kidney graft survival) OR (renal graft survival))"	28
		"((pulmonary hypertension) OR (pulmonary pressure)) AND "Kidney Transplantation"[Mesh]"	290
			Total records = 503
**Embase**	1966 to present	"((pulmonary hypertension) OR (pulmonary pressure)) AND ((kidney transplant) OR (renal transplant)) AND ((outcomes) OR (mortality) OR (survival) OR (kidney graft dysfunction) OR (renal graft dysfunction) OR (kidney graft survival) OR (renal graft survival))"	302
		"(echocardiography) AND ((pulmonary hypertension) OR (pulmonary pressure)) AND ((kidney transplant) OR (renal transplant)) AND ((outcomes) OR (mortality) OR (survival) OR (kidney graft dysfunction) OR (renal graft dysfunction) OR (kidney graft survival) OR (renal graft survival))"	64
		"((pulmonary hypertension) OR (pulmonary pressure)) AND ((kidney transplant) OR (renal transplant))"	116
			Total records = 482
**Cochrane library**	1967 to present	"((pulmonary hypertension) OR (pulmonary pressure)) AND ((kidney transplant) OR (renal transplant)) AND ((outcomes) OR (mortality) OR (survival) OR (kidney graft dysfunction) OR (renal graft dysfunction) OR (kidney graft survival) OR (renal graft survival))"	80
		"(echocardiography) AND ((pulmonary hypertension) OR (pulmonary pressure)) AND ((kidney transplant) OR (renal transplant)) AND ((outcomes) OR (mortality) OR (survival) OR (kidney graft dysfunction) OR (renal graft dysfunction) OR (kidney graft survival) OR (renal graft survival))"	6
		"((pulmonary hypertension) OR (pulmonary pressure)) AND ((kidney transplant) OR (renal transplant))"	103
			Total records = 189
**Scopus**	From the inception till present	"((pulmonary hypertension) OR (pulmonary pressure)) AND ((kidney transplant) OR (renal transplant)) AND ((outcomes) OR (mortality) OR (survival) OR (kidney graft dysfunction) OR (renal graft dysfunction) OR (kidney graft survival) OR (renal graft survival))"	622
		"(echocardiography) AND ((pulmonary hypertension) OR (pulmonary pressure)) AND ((kidney transplant) OR (renal transplant)) AND ((outcomes) OR (mortality) OR (survival) OR (kidney graft dysfunction) OR (renal graft dysfunction) OR (kidney graft survival) OR (renal graft survival))"	120
		"((pulmonary hypertension) OR (pulmonary pressure)) AND ((kidney transplant) OR (renal transplant))"	835
			Total records = 1577
All databases: 2751 records			
