# Supplementary material for: Pulmonary Arterial Hypertension and Adverse Outcomes after Kidney Transplantation: A Systematic Review and Meta-Analysis
Source: J Clin Med. 2022 Mar 31;11(7):1944. doi: 10.3390/jcm11071944 (PMC8999673; doi:10.3390/jcm11071944)
Supplement: Supplementary file 1 [file jcm-11-01944-s001.zip › Table S3 Quality assessment.pdf]

Table S3. Quality assessment of included studies using Newcastle-Ottawa scale.

Study	Representativeness of the exposed cohort	Selection of the non-exposed cohort	Ascertainment of exposure	Presence of outcome of interest at the start	Comparability of cohorts	Assessment of outcome	Follow-up long enough for outcomes to occur	Adequacy of follow-up	Total
Issa, 2008	*	*	*	*	*		*		6
Nguyen, 2021	*	*	*	*	*	*	*	*	8
Obi, 2020	*	*	*	*	*	*	*		7
Rabih, 2022	*	*	*	*	*	*	*	*	8
Sadat, 2021	*	*	*	*	*		*		6
Goyal, 2018	*	*	*	*	*	*	*		7
Wang, 2018	*	*	*	*	*	*	*		7
Zlotnick, 2010	*	*	*	*	*		*		6
Caughey, 2020	*	*	*	*	*	*	*		7
Abasi, 2020	*	*	*	*	*		*		6
Foderaro, 2017	*	*	*	*	*		*	*	7
Joseph, 2021	*	*	*	*	*		*	*	7

NA = not applicable.

Good quality: 3 or 4 stars in selection domain AND 1 or 2 stars in comparability domain AND 2 or 3 stars in outcome/exposure domain. Fair quality: 2 stars in selection domain AND 1 or 2 stars in comparability domain AND 2 or 3 stars in outcome/exposure domain. Poor quality: 0 or 1 star in selection domain OR 0 stars in comparability domain OR 0 or 1 stars in outcome/exposure domain.
